# Modern medical services, a double‐edged sword manages symptoms, but accumulates genetic background of cardiovascular diseases: A cross populational analysis of 217 countries

**DOI:** 10.1002/hsr2.1828

**Published:** 2024-01-22

**Authors:** Wenpeng You, Maciej Henneberg

**Affiliations:** ^1^ Heart and Lung Royal Adelaide Hospital Adelaide South Australia Australia; ^2^ Adelaide Medical School The University of Adelaide Adelaide South Australia Australia; ^3^ Cardiology Box Hill Hospital Melbourne Australia; ^4^ Institute of Evolutionary Medicine University of Zurich Zurich Switzerland

**Keywords:** Biological State Index (Ibs), cardiovascular diseases, gene accumulation, healthcare services, reduced natural selection

## Abstract

**Background and Aims:**

Through reduced natural selection, measured with Biological State Index (*I*_*bs*_), modern medicine enables most people to survive well beyond the reproductive lifespan leading to deleterious gene accumulation in population. This study explored the role of reduced natural selection in increasing cardiovascular disease (CVD) incidence worldwide.

**Methods:**

Country‐specific estimates of CVD incidence and the index of reduced natural selection were captured for analysis of their correlation. Aging, affluence, obesity prevalence, and urbanization were considered as the potential confounders in the analyses.

**Results:**

Worldwide, *I*_*bs*_ was significantly correlated with CVD incidence in the bivariate correlation analyses. This relationship remains when the contributing effects from aging, affluence, obesity prevalence, and urbanization are removed in partial correlation model. Multiple linear regression (enter) shows that *I*_*bs*_ is a significant predictor of CVD incidence. Stepwise multiple linear regression selects *I*_*bs*_ as the variable having the second greatest influence on CVD incidence after ageing. *I*_*bs*_ showed a significantly greater correlation with CVD incidence in low‐ and middle‐income countries (LMICs) than in high‐income countries.

**Conclusion:**

Worldwide, through reducing natural selection, the side effects of healthcare services may have been partially contributing to the increase of CVD incidence worldwide with special regard to LMICs.

## 1 BACKGROUND

Representing 32% of all global deaths, the total number of cardiovascular disease (CVD) deaths had increased to 17.9 million in 2019 from 14.4 million in 1990.^1^ It has quickly become the leading cause of death worldwide,^1^ a result which is more than 10 years earlier than previously predicted.^2^, ^3^ CVD is a life‐course and lifestyle disease, it is estimated that CVD presents over 60% of lifetime risk for global morbidity and mortality.^4^ Thus, both developed and developing countries consider the prevention of CVD a public health priority.^5^

In past decades, cardiovascular research has identified that the increasing global burden of CVD has been attributable to both genetic and environmental factors. At a population level, environmental risk factors can be integrated into four major areas; the increase of per capita income,^6^, ^7^ life expectancy,^8^, ^9^ obesity,^10^, ^11^, ^12^, ^13^ and urban lifestyle.^14^, ^15^, ^16^ Clustering CVDs in families for linkage studies has revealed that CVD pathogenesis involves strong genetic components.^17^, ^18^ For example, aggregated data of early‐onset CVD have long been attributable to kinships between family members,^19^ and this association was independent of well‐established CVD risk factors, such as smoking, dietary patterns, and physical exercise.^20^, ^21^ The genetic background predisposing to CVD may have two major sources:

1) Genes and/or mutations that directly predispose to CVDs are a group of disorders of the heart and blood vessels.
  Studies revealed that, as a group of Mendelian diseases, CVDs can be predisposed by genetic variants spanning from rare and deleterious mutations, such as familial hypercholesterolemia, to common polymorphisms modulating a predisposition to complex diseases with a weak effect for individuals.^17^, ^22^
2) Indirectly predisposed with genetically inheritable comorbidities, including, but not limited to, obesity, high blood pressure, diabetes, and chronic kidney disease.^23^

Natural selection is a key mechanism of evolution. It is a process through which populations of organisms adapt to their environments and change. Lineages that are more adapted to their environments have more opportunities to survive, participate in reproduction, and pass on the genes that contribute to their success. Human evolution involves differential factors in the survival and reproduction of individuals due to genetic differences determining phenotypes.^24^, ^25^ Because the living environments of all human populations are always subject to change, natural selection is an ongoing process and impacts each human being. Only those individuals with increased reproductive success can survive natural selection.^26^ Based on the concept of reproductive value developed by Fisher,^27^ Henneberg et al. proposed the “Biological State Index (*I*_*bs*_)” as the measure of the opportunity for an average member of a population to participate in reproduction and pass genes onto their next generation.^24^, ^25^, ^28^, ^29^, ^30^ Henneberg et al. calculated the *I*_*bs*_ by combining age‐specific death frequency (*dx*) with an age‐specific reproductive loss (*s_x_*) with the formula below^24^, ^25^, ^28^, ^29^, ^30^:

$$I_{\mathrm{bs}}=1-\sum_{x=0}^{x=\omega }\;\text{dx}\;.Sx,$$

where *dx* is the frequency of death at age *x* or represents the mortality rate. *s_x_* is the reproductive loss from dying at age *x*, that is, the estimated probability of not producing the complete number of births up to age *x. s_x_* is based on the cumulative number of births at specific ages.^25^, ^31^

Assuming that the heritability of human fertility variance is negligible,^32^ the lower level of medical services a population has, the smaller *I*_*bs*_ value it would have because of greater mortality rate. This suggests that the population faces larger effective natural selection pressure because less individuals survive to reproduce and those who survive do not use their full reproductive period to produce offspring. In such situations, detrimental phenotypes in the population may be less prevalent because the strong selection pressure reduces the passage of detrimental genes to the next generation. An *I*_*bs*_ value of 0 indicates that the population lacks the ability to overcome any selection pressure and none of the population have any opportunity to participate in reproduction. On the contrary, an *I*_*bs*_ value close to 1 indicates that natural selection is not having a significant effect on the population since almost all individuals, irrespective of their genetic endowment, are able to contribute to producing the next generation. If a population has an *I*_*bs*_ value of 1, it signifies that all members of the population have full opportunity to participate in reproduction (natural selection is totally reduced). It formally means that they are all fully adapted to their environment. Thus, the *I*_*bs*_ permits the estimation of the magnitude of the successful reproduction of a population^24^, ^25^: the role of *I*_*bs*_ has been examined in the increase of cancer,^33^ dementia,^34^ type 1 diabetes prevalence,^29^ and adult obesity prevalence.^30^, ^35^

The CVD genetic background allows individuals from a population to pass their CVD‐associated genes onto the next generation through participating in reproduction. Under a given set of mortality and fertility conditions, the fraction of a population passing CVD genetic background may be determined by the proportion of population carrying CVD genes in the next generation.

Developed populations have enjoyed high levels of medical services for 150–200 years, which has been relaxing their natural selection.^28^ This allows greater proportion of population to participate in reproduction and pass their potentially detrimental genetic background to the next generation.^28^ This has raised a public health concern that detrimental or unfavorable genetic background has been accumulated in human populations. This hypothesis has been tested by examining the role of *I*_*bs*_ in increasing the prevalence or incidence of general communicable disease,^28^ cancers,^36^ dementia,^34^ type 1 diabetes^29^ and obesity.^30^, ^35^ To further examine the role of I_*bs*_ in accumulating the detrimental genetic traits of noncommunicable diseases, country‐specific data on I_*bs*_ and CVD incidence rate were extracted for testing the hypothesis that countries with more reduced natural selection (greater *I*_*bs*_ value) may have higher CVD incidence. The major potential confounders, aging, GDP PPP, obesity, and urbanization were incorporated into the data analyses.

## 2 MATERIALS AND METHODS

### 2.1 Data sources

With reference to previously published studies,^34^, ^37^, ^38^, ^39^, ^40^, ^41^, ^42^ the country‐level data published by the United Nations and its agencies were captured for this study:

The dependent variable is the most up‐to‐date population‐specific CVD incidence rate in 2017 published by the Institute for Health Metrics and Evaluation of the University of Washington.^43^, ^44^ The estimate of CVD incidence rate is measured as the total number of people who were newly diagnosed with CVDs in 2017 per 100,000.

The independent variable is the medical‐services‐associated reduced natural selection (measured with Biological State Index [*I*_*bs*_]).^28^, ^29^, ^30^, ^33^, ^34^, ^35^

It is well‐established that CVD is a lifestyle‐associated disease^5^; however, it is known that CVD has multiple aetiologies that may be contributing to the worldwide increase in CVD incidence rate. People could develop CVD at any stage of their life. Therefore, approaches to diagnosing and preventing CVD have focused on the assessment and treatment of life‐long key risk factors. In this study, the following well‐recognized variables are included as potential confounders for analyzing the independent role of *I*_*bs*_ in CVD incidence rate:

1) Aging, indexed with life expectancy at age 65 years old in 2014 (aging, Life e_(65)_).^45^
  Published by the United Nations, the increase of life expectancy of population may be attributable to a number of factors, for example, improvements in health care, hygiene, diet and nutrition, and public health, but declining infectious disease rates.^10^ Although CVD can occur at any age, even in newborns, adults aged 65 and older are much more likely to develop CVD due to their decreasing biopsychosocial functions.^8^, ^9^
2) Gross domestic product (GDP) at purchasing power parity (PPP) per capita (GDP PPP), expressed with the total monetary value of the goods and services produced within a country in 2014 as per the World Bank.^6^, ^7^
  Gross Domestic Product PPP as a major indicator of socioeconomic status determines people's level of healthcare services that include CVD screening and the potential treatments.^7^, ^10^
3) Obesity prevalence rate, referred to the percentage of obese population in 2014 published by the WHO Global Health Observatory (GHO).^46^
  Obesity prevalence rate indicates the percentage of adult population (18 years or older) with body mass index (BMI) ≥ 30 kg/m^2^.^46^ Obesity poses multifactorial health challenges that contribute directly to the incidence of CVD risk factors, including hypertension, type 2 diabetes, dyslipidemia, and atherosclerosis in both adults and children.^10^, ^11^, ^12^, ^13^
4) Urbanization expressed with the country‐specific percentage (%) of population living in urban areas in 2014 published by the World Bank.^6^

Urbanization refers to a process in which changing to an urban lifestyle leads to a lack of physical exercise and social engagement. Despite higher levels of education and health care services, the population increases their intake of less nutritious food, gluten, processed meat, salt, fat, sugar, and alcohol, while eating less vegetables.

In the past decades, urban lifestyle has been associated with complex risk factors for chronic diseases, including CVD.^14^, ^15^, ^16^, ^37^

There are 217 “countries” reporting their health, social, and economic data to the World Bank database. Country in the World Bank database does not necessarily mean politically independent region, but a data‐reporting geographic location.^47^ We extracted the whole list of 217 countries to match the six country‐specific variables, which were saved in Microsoft Excel® 2016 for statistical analyses.

CVD as a diagnosed health condition may include the delayed presentation of risk factors such as the independent variable (*I*_*bs*_) and potential confounding variables (aging, GDP PPP, obesity, and urbanization). In other words, CVD presentation may only occur after cumulative exposure to the risk factors. For example, if a population has a larger exposure to urbanization, its CVD incidence may not expand immediately, but it may require several years of accumulated effects from the exposure. Therefore, country‐specific data on aging, GDP PPP, obesity, and urbanization were backdated 3 years to associate their prolonged confounding effects on the most recent CVD incidence data from 2017. The effects of medical service‐associated *I*_*bs*_ involve reproduction, this may require an even longer period of time for genetic background accumulation. However, considering data availability, and the delayed effects of opportunities for reproduction, data on *I*_*bs*_ calculated with United Nations fertility for 2008 and WHO mortality for 2009 were extracted to examine *I*_*bs*_ role in CVD occurrence in 2017.

### 2.2 Ethics approval and informed consent

All the data incorporated into our study were freely downloaded from the official websites of United Nations agencies and the Institute for Health Metrics and Evaluation of the University of Washington. It is not a requirement to seek ethical approval or written informed consent for applying these data for research.

### 2.3 Data analysis

We followed the data analysis approaches in the studies authored by You et al.^38^, ^39^, ^40^, ^41^, ^42^ Data analyses proceeded in five models for examining the relationship between the *I*_*bs*_ and CVD incidence.

1. The Bing© was applied to integrate the countries into the world geographic map in Microsoft Excel® 2016, and each country was colored in different levels of darkness depending on their CVD incidence rate. Intuitively, the CVD incidence rates in various countries can be visualized depending on the darkness of color and their ISO codes.
  Scatter plots were also prepared for exploring and visualizing the correlation between *I*_*bs*_ and CVD incidence at a population level. Scatter plots allow us not only to visualize the correlation between *I*_*bs*_ and CVD incidence, but also examine data quality and variable distributions.
  Raw data (not log‐transformed) were used for mapping CVD and producing scatter plots in Excel (Microsoft® 2016).
  Before running correlation analyses, all six variables were logarithmed (ln), which reduced possible curvilinearity of regressions and data non‐homoscedasticity due to their distributions.
2. Bivariate correlations (Pearson's and nonparametric) were conducted to examine the strength and direction of the correlations between all variables. This allowed us to examine the correlated variables using the common sense and previous studies. It also allowed us to check if the potential confounding variables were chosen properly.
3. Partial correlation of Pearson's moment‐product correlation was performed to examine the correlation between selection opportunity and CVD incidence while the competing variables (aging, GDP PPP, and urbanization) were kept statistically constant.
*I*_*bs*_ was first incorporated as an independent variable for predicting CVD incidence while aging, GDP PPP, obesity, and urbanization were statistically kept constant. Then *I*_*bs*_ was kept statistically constant for exploring the independent predicting effects of aging, GDP PPP, obesity, and urbanization on CVD incidence respectively.
4. Standard multivariate linear regression (enter model) was conducted to analyze the respective predicting effects of aging, GDP PPP, obesity, and urbanization on CVD incidence when we did not know which independent variables would create the best prediction equation.^48^ Subsequently, stepwise linear regression was performed to select the predictor(s) having the best‐influencing effects on CVD incidence.
  To see if and how much *I*_*bs*_ affected the predicted effects of aging, GDP PPP, obesity, and urbanization on CVD incidence in both enter and stepwise models, *I*_*bs*_ was added once and then not added as one of the predicting variables for both linear regression analyses.
5. The 217 countries were also grouped as per different classification criteria for exploring the regional relationship between *I*_*bs*_ and CVD. with nonparametric correlations:
  1) the World Bank income classifications: high income, upper middle income, low‐middle income, and low income;
    In response to the WHO's statement that at least 75% of CVD deaths occur in low‐ and middle‐income countries (LMICs),^1^ LMICs were combined to create another country grouping. Nonparametric correlation analysis was conducted to explore the correlation between *I*_*bs*_ and CVD incidence and then Fisher's r‐to‐z transformation was applied to compare the strength of correlations of *I*_*bs*_ to CVD in LMICs and high‐income countries.
  2) the developed and developing countries defined by the United Nations’ common practice^49^;
    As a further response to the above WHO statement, Fisher's r‐to‐z transformation was applied to compare the nonparametric correlations between *I*_*bs*_ and CVD incidence in developed countries and developing countries and to determine the significance of differences.
  3) the WHO regional classifications: Africa (AFR), Americas (AMR), Eastern Mediterranean (EMR), Europe (EU), South‐East Asia (SEAR), and Western Pacific (WPR)^50^;
  4) countries with the strong contrast in terms of geographic distributions, income levels, and/or cultural backgrounds. We analyzed the correlations in the eight country groupings: Asia Cooperation Dialogue (ACD)^51^; the Asia‐Pacific Economic Cooperation (APEC)^52^; the Arab World,^53^ countries with English as the official language (government websites), Latin America,^54^ Latin America and the Caribbean (LAC),^54^ the Organization for Economic Co‐operation and Development (OECD),^55^ and Southern African Development Community (SADC).^56^

Bivariate correlations (Pearson's *r* and nonparametric), Pearson's moment‐product partial correlation, and multiple linear regressions were performed with log‐transformed data in SPSS v.28. The two‐sided significance was reported when *p* was below 0.05, but the stronger significance levels, such as *p* < 0.01 and *p* < 0.001 were also indicated in this study. Regression analysis criteria were set at probability of *F* to enter ≤0.05 and probability of *F* to remove ≥0.10.

## 3 RESULTS

**Figure 1:**
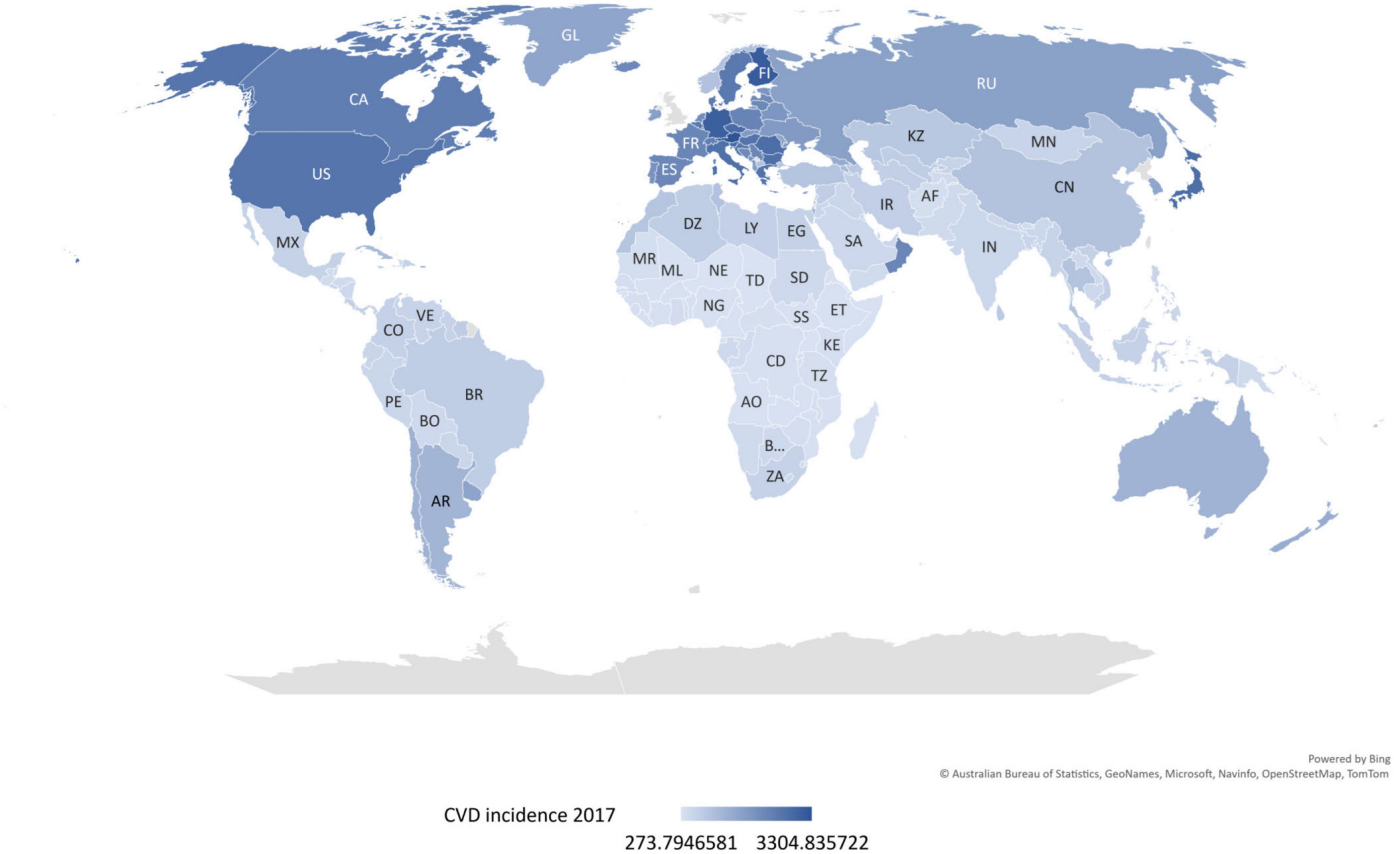
The world map to show cardiovascular disease (CVD) rates of all countries. Data source and definition: CVD incidence rate (per 100,000) 2017, University of Washington.

High CVD incidence rates occur in Europe, North America, Russia, Japan, Australia, and New Zealand (Figure 1). Empirically, they are developed countries with high levels of healthcare services. Worldwide, the highest CVD incidence rate in Austria (3304.84/100,000) was more than 12 times that in the lowest country Niger (273.79/100,000). Worldwide, the arithmetic mean of CVD incidence rate was 1017.70/100,000 (Figure 1).

**Figure 2:**
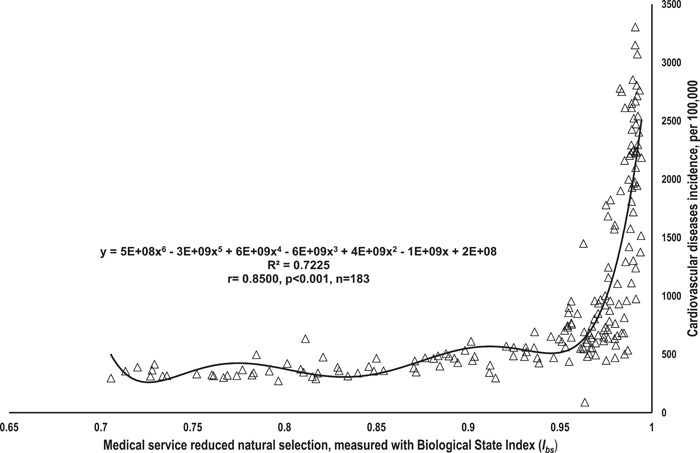
The relationship between opportunity for selection (*I*_*bs*_ *)* and cardiovascular incidence rate. Data source and definition: Biological Status Index (*I*_*bs*_), extracted from the previous study (You and Henneberg, 2018); Cardiovascular disease (CVD) incidence rate (per 100,000) in 2017, University of Washington.

Figure 2 revealed that, worldwide, *I*_*bs*_ was in a strong, positive, and significant correlation to CVD incidence rate (*R* ^2^ = 0.7225; *r* = 0.8500; *p* < 0.001, *n* = 183). There did not appear to be a major outlier in the variables of *I*_*bs*_ and CVD incidence rate.

Both variables were not log‐transformed for this correlation analysis.

Table 1 shows that, worldwide, *I*_*bs*_ was in a strong, positive, and significant correlation to variable CVD incidence in both Pearson's *r* and nonparametric analyses (*r* = 0.698, *p* < 0.001 and *r* = 0.890, *p* < 0.001, respectively). Also, in both Pearson's *r* and non‐parametric data analysis models, CVD incidence rate was in significant and moderate to strong, positive correlations to aging, GDP PPP, obesity prevalence, and urbanization respectively (*r* range: 0.428–0.890, *p* < 0.001). This suggests that the potential confounders were properly chosen.

**Table 1:** Bivariate (Pearson's *r* and nonparametric) correlation matrix between all variables.

	*I*_*bs*_	CVD Incidence	Ageing (e_(65)_)	GDP PPP	Obesity %	Urbanization
*I*_*bs*_	1	0.698^***/1.84^	0.717^***/7.57^	0.747^***/1.25^	0.529^***/2.37^	0.513^***/3.45^
CVD Incidence	0.890^***/1.84^	1	0.764^***/1.82^	0.734^***/1.19^	0.428^***/1.81^	0.526^***/1.74^
Ageing (e_(65)_)	0.871^***/7.57^	0.782^***/1.82^	1	0.811^***/1.25^	0.362^***/0.43^	0.556^***/1.25^
GDP PPP	0.863^***/1.25^	0.775^***/1.19^	0.820^***/1.25^	1	0.502^***/1.25^	0.720^***/1.25^
Obesity %	0.498^***/2.37^	0.473^***/1.81^	0.388^***/0.43^	0.483^***/1.25^	1	0.546^***/2.14^
Urbanization	0.644^***/3.45^	0.555^***/1.74^	0.604^***/1.25^	0.757^***/1.25^	0.584^***/2.14^	1

*Note*: Pearson *r* (above diagonal) and nonparametric (below diagonal) correlations are reported. Number of country range, 176–212. Significance levels: ***p < 0.001. Effect size (Cohen's *d)* is calculated based on raw data, and it is reported immediately next to *p* value. Data source and definition: Biological State Index (Ibs), extracted from the previous study (You and Henneberg, 2018); Cardiovascular disease (CVD) incidence, the number of new cases per 100,000, University of Washington; Aging indexed with life expectancy at 65 year old in 2014, United Nations; Per capita GDP PPP, measured with the per capita purchasing power parity (PPP) value of all final goods and services produced within a territory in a given year, the World Bank 2014; Obesity prevalence, measured with the percentage of population aged 18+ with BMI equal to or over 30 kg/m^2^, the World Health Organization 2014; Urbanization, measured with the percentage of population living in urban area, the World Bank 2014. All the data were log‐transformed for correlation analyses.

Table 2 suggests that *I*_*bs*_ was still a significant risk factor for CVD incidence rate while adjusting the potential confounding effects of aging, affluence, obesity, and urban advantages on CVD incidence (*r* = 0.206, *p* < 0.001). However, when *I*_*bs*_ was statistically kept constant, aging, obesity, and urbanization showed significant correlations with CVD incidence (*r* = 0.594, 0.315, and 0.528, respectively, *p* < 0.001).

**Table 2:** Partial correlation coefficients between cardiovascular disease incidence rate and predicting variables when *I_bs_* was incorporated as the independent (predicting) variable and confounder, respectively.

Variables	Partial correlation to CVD incidence			Partial correlation to CVD incidence		
	*r*	*p*	*df*	*r*	*p*	*df*
*I*_*bs*_	‐	‐	‐	0.206	<0.010/1.84	170
Ageing (e_(65)_)	0.594	<0.001/7.57	177	‐	‐	‐
GDP PPP	−0.030	0.689/1.19	179	‐	‐	‐
Obesity %	0.315	<0.001/1.81	184	‐	‐	‐
Urbanization	0.528	<0.001/1.74	180	‐	‐	‐

*Note*: Data source and definition: *Ibs* Biological State Index (*I*_*bs*_), extracted from the previous study (You and Henneberg, 2018); Cardiovascular disease (CVD) incidence, the number of new cases per 100,000 in 2017, University of Washington; Ageing indexed with life expectancy at 65 years old in 2014, United Nations; Per capita GDP PPP, measured with the per capita purchasing power parity (PPP) value of all final goods and services produced within a territory in a given year, the World Bank 2014; Obesity prevalence, measured with the percentage of population aged 18+ with BMI equal to or over 30 kg/m^2^, the World Health Organization 2014; Urbanization, measured with the percentage of population living in urban area, the World Bank 2014. All the data were log‐transformed for correlation analysis. Included as the confounding factor. Effect size (Cohen's *d*) is calculated based on raw data, and it is reported immediately next to *p* value.

Table 3 shows that, in multiple linear regression (enter), when *I*_*bs*_ was not added as a predicting variable, aging, and GDP PPP were the only two variables having a significant influence on CVD incidence. When *I*_*bs*_ was added as a predicting variable, *I*_*bs*_, aging and GDP PPP were the only three variables having a significant influence on CVD incidence. Regardless of *I*_*bs*_ being a predicting variable or not, obesity and urbanization showed nearly nil correlation to CVD incidence.

**Table 3:** Multiple linear regression analyses (enter and stepwise) were conducted to demonstrate that *I*_*b*s_ is a significant predictor of cardiovascular disease incidence rate.

Enter	*I*_*bs*_ not added		Enter	*I*_*bs*_ added	
Variables entered	Beta	Significance	Variables entered	Beta	Significance
*I*_*bs*_	Not added		*I*_*bs*_	0.207	<0.010/1.84
Ageing (e_(65)_)	0.501	<0.001/7.57	Ageing (e_(65)_)	0.424	<0.001/7.57
GDP PPP	0.334	<0.001/1.19	GDP PPP	0.249	<0.050/1.19
Obesity %	0.074	0.197/1.81	Obesity %	0.022	0.713/1.81
Urbanization	−0.056	0.437/1.74	Urbanization	−0.033	0.647/1.74

Stepwise	*I*_*bs*_ not added		Stepwise	*I*_*bs*_ added	
Rank	Variables entered	Adjusted *R* ^2^	Rank	Variables entered	Adjusted *R* ^2^
1	Ageing (e_(65)_)	0.593	1	Ageing (e_(65)_)	0.587
2	GDP PPP	0.630	2	*I*_*bs*_	0.626
	*I*_*bs*_	Not added	3	GDP PPP	0.639
	Obesity %	Insignificant		Obesity %	Insignificant
	Urbanization	Insignificant		Urbanization	Insignificant

*Note*: Data source and definition: Biological State Index (*I*_*bs*_), extracted from the previous study (You and Henneberg, 2018); Cardiovascular disease (CVD) incidence, the number of new cases per 100,000 in 2017, University of Washington; Ageing indexed with life expectancy at 65 years old in 2014, United Nations; Per capita GDP PPP, measured with the per capita purchasing power parity (PPP) value of all final goods and services produced within a territory in a given year, the World Bank 2014; Obesity prevalence, measured with the percentage of population aged 18+ with BMI equal to or over 30 kg/m^2^, the World Health Organization 2014; Urbanization, measured with the percentage of population living in urban area, the World Bank 2014. All the data were log‐transformed for correlation analysis. Effect size (Cohen's d) is calculated based on raw data, and it is reported immediately next to *p* value.

Subsequently, stepwise multiple linear regression revealed that aging and GDP PPP were placed first and second most significant predicting variables for CVD incidence when *I*_*bs*_ was not added as a predicting variable. When *I*_*bs*_ was added as a predicting variable, it was placed second most influential variable on the CVD incidence (increasing *R* ^2^ from 0.626 to 0.639). Aging and GDP PPP were placed first and third most influential predictors for CVD incidence.

In both enter and stepwise models, obesity and urbanization seemed not that powerful in predicting CVD incidence.

Table 4 indicates that, although countries were grouped with various criteria, *I*_*bs*_ is always in positive correlation to CVD incidence rate. However, the strengths and significance levels of *I*_*bs*_‐CVD incidence relationships varied. It is worth highlighting that *I*_*bs*_ was a significantly stronger correlation to CVD incidence in LMICs than in high‐income countries (*z* = 3.62, *p* < 0.001). Similarly, *I*_*bs*_ was a significantly stronger correlation with CVD incidence in developing countries than in developed countries (*z* = 4.33, *p* < 0.001).

**Table 4:** Nonparametric correlations coefficients between *I*_bs_ and cardiovascular disease incidence rate in different country groupings.

Country groupings	Nonparametric “rho”	*p*/Cohen's *d*	*n*
Worldwide	−0.890	<0.001/1.84	183
The World Bank income classifications			
High Income	0.587	<0.001/3.10	56
Low Income	0.324	0.099/5.94	27
Low Middle Income	0.858	<0.001/2.75	47
Upper Middle Income	0.546	<0.001/2.47	53
Low‐ and middle‐income countries (LMICs)	0.853	<0.001/2.14	127
Fisher r‐to‐z transformation	LMIC versus High: *z* = 3.62, *p*<0.001		
United Nations common practice			
Developed	0.402	<0.010/4.93	44
Developing	0.833	<0.001/2.53	139
Fisher r‐to‐z transformation	Developing versus Developed: *z* = 4.33, *p* < 0.001		
WHO regions			
Africa	0.474	<0.001/4.12	45
Americas	0.753	<0.001/2.57	34
Eastern Mediterranean	0.616	<0.010/2.36	21
Europe	0.559	<0.001/3.89	51
South‐East Asia	0.370	0.293/5.33	10
Western Pacific	0.801	<0.001/2.30	22
Countries grouped with various factors			
Asia Cooperation Dialogue	0.686	<0.001/2.44	33
Asia‐Pacific Economic Cooperation	0.768	<0.001/2.44	19
Arab World	0.596	<0.010/2.21	21
English as Official Language	0.876	<0.001/1.93	50
Latin America	0.866	<0.001/3.12	20
Latin America and Caribbean	0.707	<0.001/3.80	32
Organization for Economic Co‐operation and Development	0.503	<0.010/4.10	37
Southern African Development Community	0.603	<0.050/3.74	16

*Note*: Nonparametric correlations within country groupings were reported. Data source and definition: Ibs Biological State Index (*I*_*bs*_), extracted from the previous study (You and Henneberg, 2018); Cardiovascular disease (CVD) incidence, the number of new cases per 100,000 in 2017, University of Washington. All the data were log‐transformed for correlation analysis. Effect size (Cohen's *d*) is calculated based on raw data, and it is reported immediately next to *p* value.

## 4 DISCUSSION

CVD is a leading risk factor for disability and death worldwide which significantly contributes to the escalating costs of healthcare services in managing morbidity and mortality. By examining the correlation between medical services associated *I*_*bs*_ and CVD incidence data at population level, the findings of this study suggest that:

1. As a side effect of modern medicine, reduced natural selection (*I*_*bs*_) may have become a significant determinant of increasing global burden of CVD incidence.
2. The *I*_*bs*_ has a statistically significant relation to CVD pathogenesis which is independent of the potential confounding effects of aging, GDP PPP (affluence), obesity prevalence, and urbanization on CVD incidence.
3. Once the combined effects of *I*_*bs*_, aging, GDP PPP, obesity, and urbanization are all considered, the correlations between CVD incidence and both obesity and urbanization statistically disappear, indicating that the exposure of *I*_*bs*_, aging, and GDP PPP may be able to explain why obesity and urbanization have been correlated with CVD incidence rates.
4. Increased income correlates with increased overall medical services available among LMICs due to their emerging economies and significant improvements in medical care. This may be reflected by the significantly stronger correlation between *I*_*bs*_ and CVD in LMICs than in developed countries.

Globally, medical science has developed significantly in the past couple of hundred years. The wide use of medicine has been reducing natural selection in contemporary populations.^28^ Such medical intervention frequently allows individuals to survive and reproduce.^57^ In other words, the probability for an average person from each country to survive their reproductive lifespan has increased. It was empirically estimated that, 150 years ago, the probability for individuals born into a population to pass their genes to the next generation was less than 50% because of a low level of healthcare services.^58^, ^59^ Indexed by *I*_*bs*_,^24^, ^25^ the worldwide average probability for an individual to participate in reproduction and pass their genetic background is 92.8%, and countries with the highest and lowest probabilities for each individual to participate in reproduction are Cyprus and Iceland (99.4%) and Burkina Faso (63.5%), respectively.^29^, ^30^, ^33^, ^35^ None of the countries has an *I*_*bs*_ equal to 1, that is, 100%,^28^, ^29^, ^30^, ^33^, ^34^, ^35^ so natural selection still acts on members of each population and adjusts gene frequency variations in each population through the action of differential fertility and mortality over time.^60^ The magnitude of the reduction in natural selection may differ between countries due to the level of medical services, which include not only the direct clinical interventions, such as diagnosis and treatment, but also those nonclinical interventions, such as sanitation, prevention of disease and socioeconomic position (as reflected by income, education, and occupation).^28^

Primarily, natural selection acts as the “janitor of the gene pool” eliminating deleterious mutations which make individuals unfit for surviving their environments.^36^ Fertility and mortality operate singly or jointly to determine the level of fitness of a particular population in a given environment because it measures reproductive success.^60^ As the basic events of natural selection,^60^ country‐specific differential fertility and mortality rates have been integrated into the formula for calculating their Biological State Index (*I*_*bs*_),^24^, ^25^, ^28^, ^29^, ^30^ which indicates their different successful reproduction opportunities of individuals.^28^ The opportunity for successful reproduction in individuals of a population may determine the magnitude of heritable CVD genes to be passed onto the next generation influencing their CVD incidence. In the present study, the positive correlation between *I*_*bs*_ and the CVD incidence rate has been observed. This suggests that a lower opportunity for natural selection allows the accumulation of deleterious genes, including CVD genes.^28^, ^61^

With polymorphic DNA markers throughout the genome, linkage studies and genome‐wide linkage analyses have found that polygenic backgrounds have been associated with the majority of CVDs and CVD risk factors.^17^, ^18^ CVDs have a large number of genetic variants which span from rare and deleterious mutations to common polymorphisms modulating the predisposition to complex diseases.^17^, ^22^ Although some CVDs can be fatal, CVD phenotypes do not develop in the majority of genetically predisposed populations which may be attributable to clinical and nonclinical healthcare interventions. This enables the genetic predisposition of CVDs to keep accumulating in human populations until persons develop CVDs and die. While specific genes predisposing to specific CVDs may still remain unknown, the strong statistical relationship between *I*_*bs*_ and CVD incidence is clear (*r* = 0.7122, *p* < 0.001). This suggests the general tendency that relaxed natural selection allows detrimental genetic background to accumulate, especially if single detrimental alleles have mild effects.^62^ Previous evolutionary research has suggested that phenylketonuria (an inheritable disease) only became noticeable after the genetic background has accumulated for several generations^28^ with about a 2% increase each generation due to reduced natural selection.^57^

Currently, the focus of clinical interventions for CVDs and the comorbidities is their symptoms, but not the genetic background that determines the CVD phenotypes. Clinically, when patients are “cured” from their health conditions of CVDs and their comorbidities, it only indicates that their symptoms are under control, instead of their genetic background (genes/mutation) and other causes being controlled. While symptoms may be controlled, these CVD patients who carry a CVD genetic background can still reproduce and pass their CVD genetic background onto the next generation, leading to an accumulation of CVD in the human population.

CVD gene therapy aims to modify monogenetic traits and polygenic backgrounds of CVDs to provide therapeutic effects for preventing, treating, and eventually reducing CVD in the human population. However, the therapy progresses very slowly because of technical defects, such as vectors and delivery to the target cell and ethical issues.^63^, ^64^ Therefore, at present, the therapy has not been operational in clinical settings.^63^, ^64^

In this study, we took a quantitative approach to examining the side effects of modern medicine on CVDs. This is essentially different from previous studies about the relationship between CVD and natural selection. Ding and Kullo conducted a systematic review outlining several important medical advances that may be able to locate the CVD gene variants due to natural selection forces for exploring the subsequent gene therapy.^65^ Alongside the effects of familial hypercholesterolemia on CVDs in the 19th century, Poledne and Zicha described how people were genetically adapted to high levels of sodium diet which acted as a selection force.^66^ The result from this selection was that the level of capability to retain sodium determined the opportunity for people to survive.^66^ From the public and global health perspective, Corbett et al. raised a concern that the direction and intensity of natural selection may have been diversified due to complex modernity.^67^ Several studies conducted at molecular level, such as SNP arrays, genome sequencing, or case–control comparisons, controversially reported the correlations between high‐altitude (a selection force) and hypoxia‐signaling‐pathway‐related genes across Andean highlander genomes.^68^, ^69^, ^70^, ^71^ Obviously, these previous studies, which have not shown clinical significance, did not suggest that the more advanced medicine is, the more likely it reduces natural selection allowing the CVD‐associated genetic background to accumulate in human population.

The positive correlations of *I_b_*_*s*_ with CVD incidence were also observed in different country groupings sharing specific characteristics such as per capita income level, geographic locations, cultural backgrounds, and affiliations to international functional organizations. In this study, the correlation between *I_bs_* and CVD incidence is significant and strong in LMICs (*r* = 0.853, *p* < 0.001), which is significantly stronger than the correlation in high‐income countries (*z* = 3.62, *p* < 0.001) (Table 4). This may be attributable to the emerging economies in LMICs where medical services have improved increasing the variance of *I*_*bs*_. In LMICs, the impact of an emerging economy may, ironically, have led to high level of medical services while reducing natural selection faster than previously thought. In our data analysis, *I*_*bs*_ showed a significantly stronger correlation in LMICs than in high‐income countries. These latter countries may have a smaller variability of *I_bs_* and CVD that automatically lowers covariance.

### 4.1 Strengths and limitations

It is clear that *I_bs_* predicts CVD incidence significantly and independently both worldwide and with special regard to LMIC. However, there are several important study limitations to consider.

1) This is an ecological study that is subject to ecological fallacy, that is, correlation at population level may not necessarily be true at individual level. To minimize the risk of erroneous correlation, with the advantages of ecological studies in accessing more variables, we adopted a number of potential confounding variables, but this did not eliminate intrinsic bias in observational studies, “correlation is not causation.”
2) The results of this study cannot be traced at individual level as natural selection is about genetic variation at the population level. This is a cross‐sectional study, but reduced natural selection is a longitudinal phenomenon. With the United Nations publishing the fertility and mortality data for more years in the future, more *I*_*bs*_ across longer periods of time would be able to be calculated for longitudinal correlation of the predicting effects of reduced natural selection on CVD incidence increase.
3) The population level data extracted from IHME, WHO, United Nations, and World Bank for this study might be fairly crude because they are the estimates of a broader population instead of the entire population. Additionally, human errors may have been made in the collection and aggregation of the data.
4) From a Darwinian evolutionary perspective, the opportunity for natural selection is only measured here with respect to postnatal mortality, which does not include gametic selection and intrauterine mortality.^59^

## 5 CONCLUSION

Worldwide, modern medicine may have been reducing natural selection and subsequently increasing the probability for an average person in a population to pass their affected genes to the next generation. Natural selection can effectively act on CVDs because of their strong genetic background. This study suggests that countries with greater levels of medical services may have higher CVD incidence rates because of reduced natural selection. This was evidenced both worldwide and with special regard to LMICs.

## AUTHOR CONTRIBUTIONS

**Wenpeng You**: Conceptualization; data curation; formal analysis; funding acquisition; investigation; methodology; project administration; resources; software; validation; visualization; writing—original draft. **Maciej Henneberg**: Conceptualization; data curation; formal analysis; funding acquisition; investigation; methodology; resources; supervision; writing—review and editing.

## CONFLICT OF INTEREST STATEMENT

The authors declare no conflict of interest.

## TRANSPARENCY STATEMENT

The lead author Wenpeng You affirms that this manuscript is an honest, accurate, and transparent account of the study being reported; that no important aspects of the study have been omitted; and that any discrepancies from the study as planned (and, if relevant, registered) have been explained.

## Data Availability

All the data are secondary and their sources have been described in Section 2. The data included in this study are published by international organizations on their official websites. There is no requirement for us to seek a formal permission before downloading and analyzing the data because of our academic research purpose.
